# Development of an In-House Assistant Application to Reduce Preparation Time of Preoperative Informed Consent Forms for Anesthesia

**DOI:** 10.7759/cureus.110998

**Published:** 2026-06-16

**Authors:** Masaki Takekoshi, Kunihiro Mitsuzawa, Takashi Ishida, Aiba Kazuma, Tanaka Satoshi

**Affiliations:** 1 Department of Anesthesiology and Resuscitology, Shinshu University School of Medicine, Matsumoto, JPN

**Keywords:** anesthesiology, assistant application, electronic medical record, informed consent form, streamlining administrative tasks, workflow efficiency

## Abstract

Introduction

Documentation-related tasks, including preparation of informed consent (IC) forms for anesthesia, increase workload and cognitive burden in anesthesiology. This study evaluated the time-saving effect and usability of a semi-automated institution-specific assistant application for anesthesia IC form preparation.

Methods

We developed a semi-automated Python-based (Python Software Foundation, Wilmington, DE, USA) assistant application using generative AI assistant programming. The application was tailored to our institutional electronic medical record (EMR) workflow and was designed to automatically select relevant checkboxes and insert required text into IC forms for anesthesia. Its time-saving effect and usability were evaluated using a randomized crossover design. Twenty anesthesiologists were randomized into two groups: Group A (n = 10, 50%) prepared IC forms for five mock patients first by manual entry and then using the application, whereas Group B (n = 10, 50%) followed the reverse order. Preparation time was defined as the interval from the start of preparing the first IC form to the completion of the fifth IC form. Usability was assessed using a 5-point Likert scale.

Results

The mean preparation time was 9.7 (SD 1.7) minutes with the application and 16.0 (SD 2.6) minutes without the application (mean difference, 6.2 minutes; 95% CI, 5.2 to 7.2 minutes; p < 0.001). Use of the application reduced preparation time by approximately 40%. Regarding usability, 11 participants (55%) reported that they “very much wanted to continue using” the application, whereas nine participants (45%) reported that they “wanted to continue using” it.

Conclusion

The tailored application significantly reduced preparation time compared with manual entry and was well accepted by participants. These findings provide proof-of-concept evidence that generative AI-assisted in-house development may enable clinicians to create institution-specific workflow-support tools for improving documentation efficiency in anesthesiology practice.

## Introduction

Physicians are required to perform various administrative tasks, including preparation of medical documents, resulting in substantial time being spent on these activities rather than on direct patient care [1,2]. Frequent handling of medical documentation increases cognitive load [3]. In anesthesiology, preoperative assessment includes reviewing patient information, determining anesthetic management, and preparing informed consent (IC) forms. Although these documentation tasks are important for patient safety and standardized perioperative communication, the associated manual transcription and repetitive data-entry processes can be time-consuming and burdensome.

At many institutions, limitations in interoperability and customization within existing hospital information systems (HIS) and electronic medical record (EMR) systems often require anesthesiologists to perform repetitive administrative tasks, such as redundant manual data entry and checkbox selection. These system-related workflow inefficiencies may increase workload and reduce the efficiency of document preparation. In particular, at our institution, preparation of individualized IC forms for each patient is administratively burdensome.

One possible solution is to develop in-house applications that complement existing systems and are adapted to local workflows, such as large language model-based applications [4-6]. Python (Python Software Foundation, Wilmington, DE, USA) is one of the most widely used programming languages in the world and is often used for developing assistant applications to improve the user interface of EMR systems [7]. In addition, recent advances in generative AI may enable clinicians with limited programming experience to develop customized applications tailored to local operational needs [8]. In the anesthesiology department, assistant applications for streamlining the preparation of individualized IC forms for each patient may reduce the time required for documentation tasks [9]. However, few prospective studies have evaluated interventions aimed at reducing the preparation time associated with anesthesia-related documentation tasks [9]. Therefore, we developed a semi-automated assistant application integrated with our institution's EMR system using Python with AI-assisted programming and investigated its time-saving effect on the preparation of IC forms as well as its usability using a questionnaire.

## Materials and methods

This randomized crossover study was approved by the Shinshu University School of Medicine Ethics Committee for Research in Life Sciences and Medicine (approval No. 6362). It was registered in the University Hospital Medical Information Network Clinical Trial Registry (UMIN000056300).

We developed an assistant application that assists in the preparation of IC forms for anesthesia. This application was developed using Python as the programming language and Visual Studio Code (Microsoft®, Redmond, WA, USA) as the code editor on a HIS terminal running EMR software. We created the application’s code with the assistance of generative AI (ChatGPT-4o; OpenAI, San Francisco, CA, USA) through an interactive, dialogue-based pair-programming approach, in which the AI served as a guiding partner, as the authors have limited coding experience. The medical information department of our institution approved the use of a local, offline application development environment to minimize the risk of personal information leakage.

**Figure 1:**
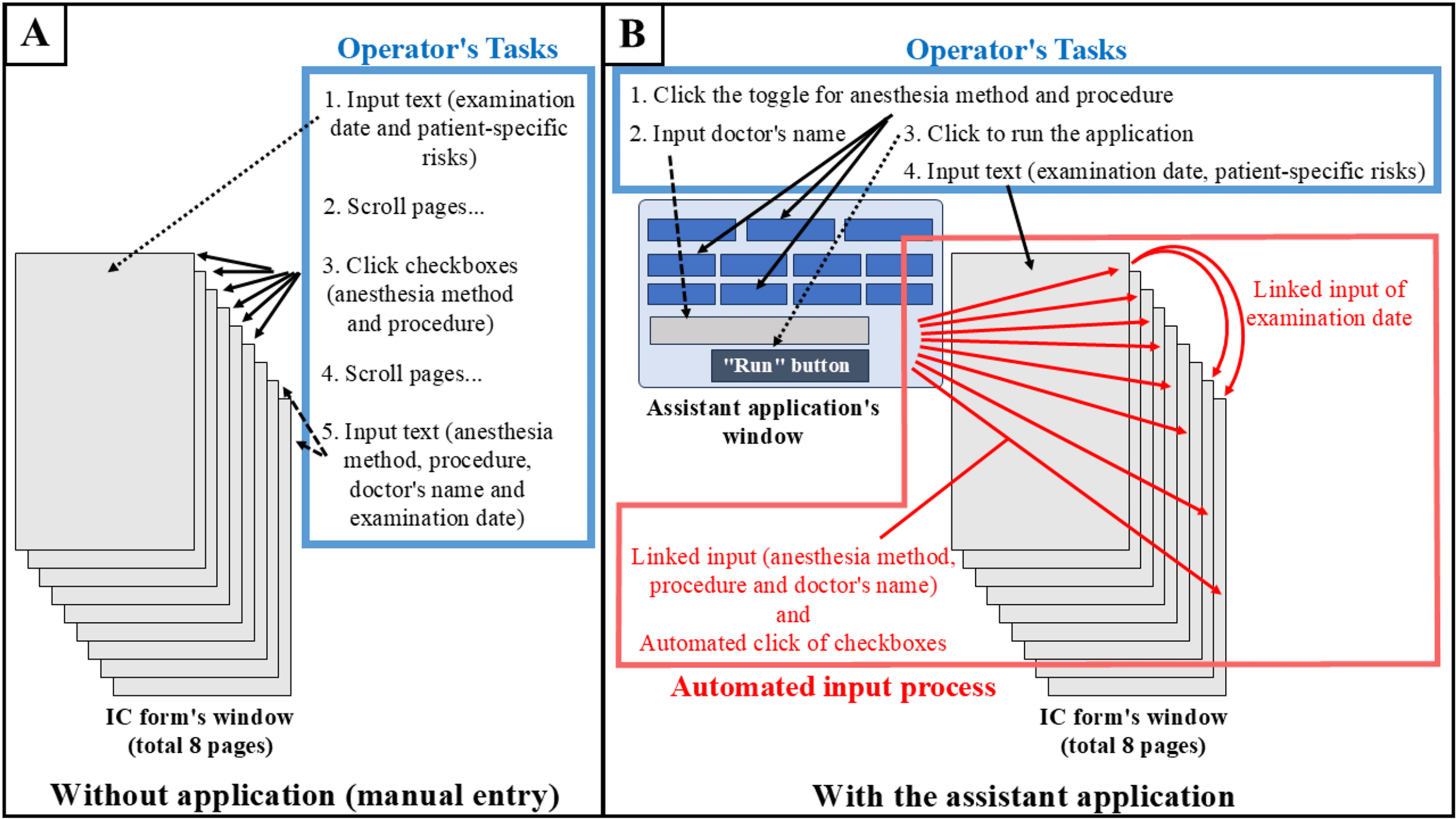
Overview of IC form creation A: An illustration of the manual method where the operator completes the required items in the IC form, including anesthesia methods, invasive procedures, and scheduled examinations, by checking boxes and entering text across multiple pages. The operator must also sign or enter their name in two designated locations. B: An illustration of the assistant application where a selection window allows the operator to choose items to be included in the anesthesia IC form. By pressing the 'Run' button, the selected items are automatically entered into the appropriate sections of the IC form. This figure was created by author Masaki Takekoshi, using Microsoft PowerPoint (Microsoft®, Redmond, WA, USA). IC: Informed consent

At our institution, IC forms must include descriptions of the anesthesia method, invasive procedures, and scheduled examinations such as general anesthesia, central venous catheterization, and transesophageal echocardiography. Currently, these elements are completed by manually checking boxes and entering text in the IC form; however, because they are distributed across multiple pages, completing the form is time-consuming. To streamline this workflow, the assistant application provides a new selection window listing explanatory items, automatically selects the corresponding checkboxes, and inserts the required text into the IC form. These functions were programmed to match the institution-specific configuration of the HIS. The operator only needs to select the appropriate items on the application, start the automation process, print the form, and affix their personal seal in two designated locations (Figure 1).

To evaluate the effectiveness of the application, EMRs for five mock patients were prepared, each containing the information necessary for creating the IC forms. The required content of the IC forms, including details of anesthesia methods, invasive procedures, and examinations, was predefined and recorded in the EMR.

This randomized crossover study enrolled 20 anesthesiologists from our institution. None of the participants were involved in the development of the application. All participants received instructions for using the application and reviewed mock patient information in the EMR in advance. The participants were allocated to two groups (Group A and Group B) using block randomization. In Group A, participants first prepared IC forms for five mock patients without the application and then prepared IC forms for the same patients using the application. In Group B, the order was reversed. Preparation time for the anesthesia IC forms was defined as the interval from the start of preparing the first patient’s IC form to the completion of the fifth patient’s IC form.

Participants completed a survey to assess their intention to continue using the application and its usability. The survey consisted of two questions, each rated on a 5-point Likert scale. Question one assessed whether the participant would like to continue using the application in daily clinical practice, i.e., (1) very much wants to use it; (2) wants to use it; (3) is neutral; (4) does not want to use it much; and (5) does not want to use it at all. Question two assessed whether the participant thought the application was easy to use: (1) very easy to use; (2) easy to use; (3) neutral; (4) not easy to use; (5) difficult to use. The primary outcome was the time required to prepare anesthesia IC forms for five mock patients, and the secondary outcome was the survey results. We also conducted a subgroup analysis of preparation time based on whether participants were board-certified anesthesiologists of the Japanese Society of Anesthesiologists or anesthesiology residents.

We predicted that the application would reduce participants' preparation time by an average of 20% compared with manual entry. A power analysis was performed with an SD estimated at 30% of the mean preparation time, a significance level of 0.05, and a power of 0.8. This calculation indicated that 20 participants were required. Data are presented as means (SD) or percentages. For the primary outcome, preparation times with and without the application were compared using a paired t-test. A linear mixed-effects model was applied to examine potential period and carryover effects. A p-value < 0.05 was considered significant. Statistical analyses were conducted using SPSS Statistics version 26 (IBM Corp., Armonk, NY, USA), and the graph for the main outcome was created using R version 4.5.1 (R Foundation for Statistical Computing, Vienna, AUT).

## Results

**Figure 2:**
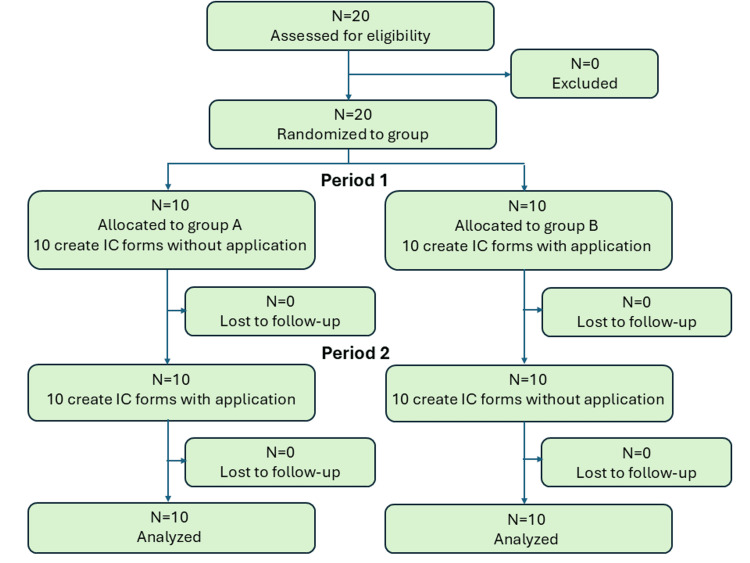
Consolidated Standards of Reporting Trials (CONSORT) flow diagram This flowchart depicts the path taken by participants through the trial. IC: Informed consent

A total of 20 anesthesiologists participated in this study. Of these, 10 were board-certified anesthesiologists (50%), and 10 were residents (50%) (Figure 2).

**Figure 3:**
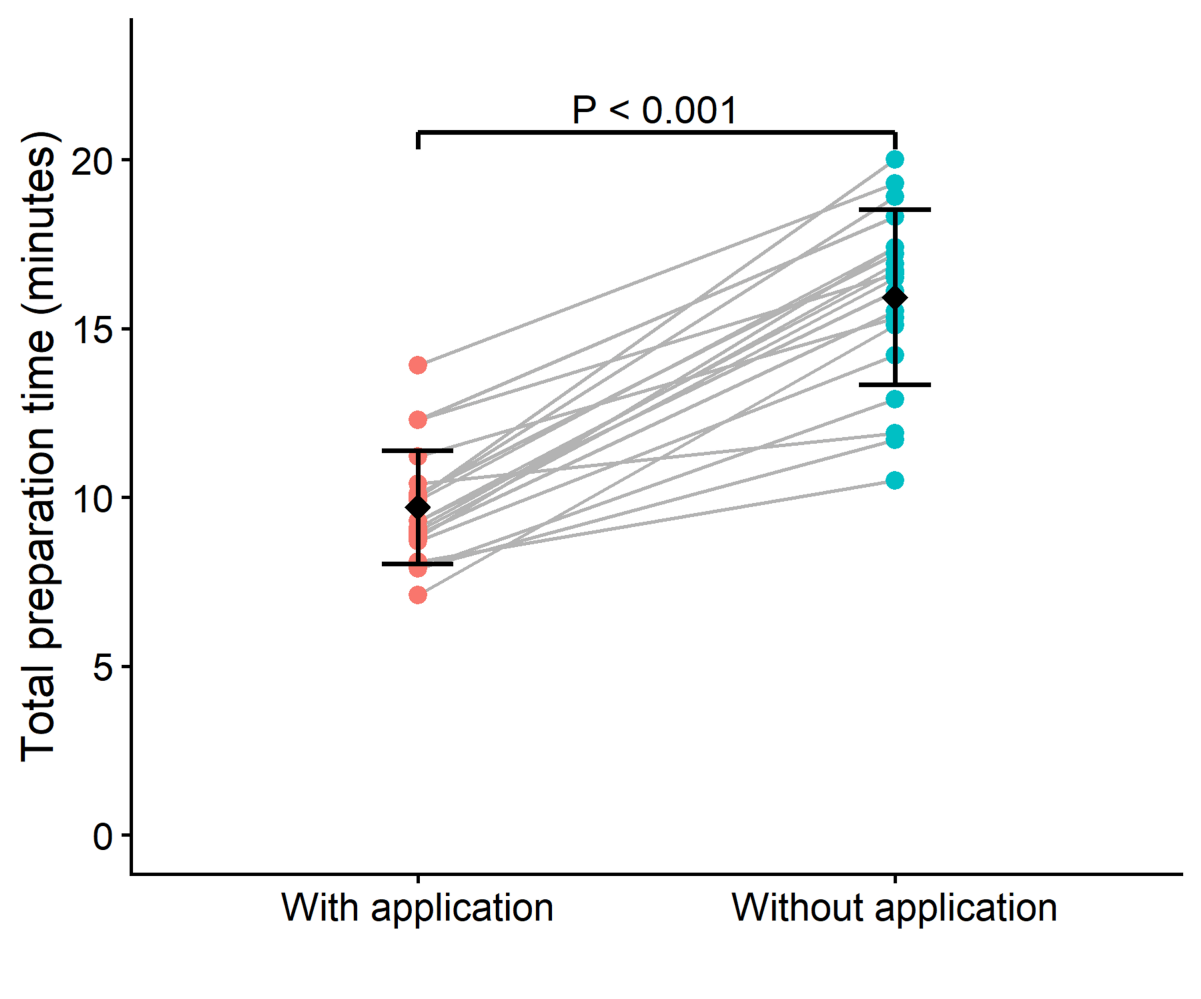
Paired dot plots of the total time required to create IC forms for five mock patients (preparation time) with and without the assistant application Error bars indicate mean ± standard deviation; preparation time was significantly shorter when using the assistant application; *p < 0.001 via paired t-test IC: Informed consent

Total preparation time (SD) of IC forms for five mock patients was 9.7 (1.7) minutes with the application and 16.0 (2.6) minutes without the application. Preparation time was significantly reduced by an average of 40% with the application compared with manual entry (mean difference, 6.2 minutes; 95% CI, 5.2 to 7.2 minutes; p < 0.001) (Figure 3).

In subgroup analyses, a significant reduction in preparation time was observed in both the board-certified anesthesiologists (mean difference, 6.8 minutes; 95% CI, 5.4 to 8.1 minutes; p < 0.001) and residents (mean difference, 7.3 minutes; 95% CI, 6.2 to 8.4 minutes; p < 0.001). No significant period or carryover effects were observed (p = 0.576 and p = 0.276, respectively). Regarding user satisfaction, 11 participants (55%) responded that they 'very much want to use it,' and nine participants (45%) responded that they 'want to use it' to the question on continued use in daily practice. Furthermore, 11 participants (55%) rated the application as 'very easy to use,' and nine participants (45%) rated it as 'easy to use.'

## Discussion

Improving workflow efficiency is an increasingly important issue in healthcare systems facing workforce shortages and growing administrative demands [10,11]. In anesthesiology, preoperative assessment involves reviewing patient information, determining anesthetic management, and preparing IC forms. Although IC itself remains a core professional responsibility of anesthesiologists, the documentation process often contains repetitive and standardized components that may be suitable targets for workflow-support interventions.

In our institution, preparation of IC forms for anesthesia requires repetitive manual data entry across multiple pages within the EMR system and is considered a substantial administrative burden. In this study, use of the assistant application significantly reduced preparation time of IC forms for anesthesia by both board-certified anesthesiologists and residents. These findings suggest that the application may streamline repetitive documentation tasks regardless of clinical experience level. The observed time-saving effect was likely attributable to reduced repetitive manual tasks, including scrolling through pages, selecting checkboxes, and entering standardized text.

Commercial EMR systems may be difficult to customize according to institution-specific workflows [12], and limited interoperability between hospital information systems may necessitate redundant manual data entry. In our setting, these limitations created a need for a locally adapted workflow-support solution. The application developed in this study was designed specifically for our institutional workflow and enabled semi-automated completion of repetitive documentation tasks.

An important aspect of this study is that the application was developed in-house using generative AI-assisted programming despite the authors’ limited coding experience, facilitated by recent advances in generative AI [10]. The novelty of this study lies not in the concept of automated form completion itself, which is already available in some EMR systems, but rather in demonstrating the feasibility of clinician-led development of institution-specific workflow-support tools using AI-assisted programming. This approach may provide clinicians with a practical means of addressing local operational challenges in healthcare environments where commercial systems cannot sufficiently accommodate institution-specific needs.

In addition to software-based approaches, another possible strategy for reducing administrative burden is the use of medical scribes [13]. However, this approach requires additional personnel and financial resources, whereas AI-assisted in-house development may represent a potentially resource-efficient strategy for supporting institution-specific documentation workflows.

This study has several limitations. First, it was conducted at a single institution in a simulated environment using mock patients; therefore, the external validity and generalizability of the findings may be limited. Because the study did not involve actual clinical workflow, the results may not fully reflect real-world clinical environments, including interruptions, workflow complexity, and cognitive demands encountered in routine anesthesiology practice. In addition, institutional differences in EMR configuration, workflow structure, and information security policies may affect the feasibility and effectiveness of similar applications in other settings. Second, usability was assessed using only two non-validated questionnaire items and therefore represents a preliminary rather than comprehensive evaluation. Future studies should incorporate qualitative assessments and established usability instruments such as the System Usability Scale (SUS). Third, although participants reviewed and corrected the generated IC forms, this study did not independently evaluate documentation accuracy, completeness, error rates, or safety-related outcomes. Furthermore, excessive reliance on semi-automated systems may lead to reduced vigilance or 'automation complacency' and increase the risk of documentation errors [14,15]. Therefore, physician review and verification of generated content remain essential even when using workflow-support applications. Finally, the application was specifically designed for our institutional EMR environment, which may limit direct reproducibility in other healthcare systems. Accordingly, the findings of this study should be interpreted as proof-of-concept evidence obtained in a controlled simulated setting rather than definitive evidence of broader clinical effectiveness.

## Conclusions

We developed a semi-automated assistant application for anesthesia IC form preparation using Python and generative AI-assisted programming. The application, which was tailored to our institutional EMR workflow, significantly reduced preparation time compared with manual entry and was well accepted by participants. These findings suggest that generative AI-assisted in-house development may enable clinicians to create institution-specific workflow-support tools and improve documentation efficiency in anesthesiology practice.
